# Esophageal Pseudotumor Secondary to Treatment with a Potassium Binder Resin: A Case of Severe Esophagitis Mimicking a Malignancy

**DOI:** 10.1155/2022/1329038

**Published:** 2022-02-27

**Authors:** Luis Chavez, Marco Bustamante-Bernal, Osvaldo Padilla, Jose Gavito-Higuera, Marc Zuckerman

**Affiliations:** ^1^Department of Internal Medicine, Texas Tech University Health Sciences Center, El Paso, TX, USA; ^2^Division of Gastroenterology, Texas Tech University Health Sciences Center, El Paso, TX, USA; ^3^Department of Pathology, Texas Tech University Health Sciences Center, El Paso, TX, USA; ^4^Department of Radiology, Texas Tech University Health Sciences Center, El Paso, TX, USA

## Abstract

*Background*. Sodium polystyrene sulfonate is a resin used to treat hyperkalemia. Colonic mucosal injury, intestinal ischemia, necrosis, and perforation have been widely reported in the literature, but few cases have reported upper gastrointestinal injury and identify the endoscopic features. *Case Presentation*. We describe a case of an 83-year-old male, with no prior esophageal symptoms, who developed dysphagia after being treated with sodium polystyrene sulfonate for hyperkalemia. Endoscopic features consistent with severe esophagitis and a mass in the lower esophagus mimicking a malignancy were found, and pathology confirmed resin-induced esophagitis. *Discussion*. The identification of basophilic crystals in the epithelium with surrounding inflammation is a hallmark of sodium polystyrene sulfonate-induced mucosal injury. Several direct and indirect mechanisms by which SPS may cause mucosal injury have been identified. Prolonged stasis of crystals in the lumen has the potential of developing erosions and ultimately necrosis. The internalization of these crystals to the underlying intestinal mucosa with the combination of the inflammatory response may give an appearance of a luminal mass that can mimic a malignancy. Recognizing the wide-ranging endoscopic findings of resin-induced mucosal injury in the esophagus is fundamental to consider a potential side effect of sodium polystyrene sulfonate. The use of this resin should be avoided in patients with suspected esophageal motility disorder.

## 1. Introduction

Sodium polystyrene sulfonate (SPS) is a commonly used resin to treat hyperkalemia. This medication exchanges sodium cations for hydrogen ions during the passage through the gastrointestinal (GI) tract. In doing this, hydrogen ions are exchanged for potassium and excreted with the modified resin [1]. SPS became popular after the 1960s, and several GI complications were reported after its increased utilization [2]. A recently published large retrospective study reported a higher risk of serious adverse GI events in hospitalized patients who received SPS [3]. The colon is the most commonly affected segment of the GI tract. Patients with prolonged use of potassium-binding resins who have chronic constipation or a mechanical obstruction may develop colonic necrosis due to long exposure of the resin to mucosa. Usually, the endoscopic lesions are ulcerations and erosions, although a few case reports have documented mass-like lesions in the colon mimicking malignancy [4, 5]. Well-known risk factors for GI complications are uremia, end-stage renal disease, hemodynamic instability, kidney transplantation, gastrointestinal surgery, and constipation [3]. The upper GI tract is less affected due to a shorter duration of resin exposure; however, esophageal and gastric lesions have been identified in patients with risk factors [6–8]. We present a case of resin-induced severe esophagitis mimicking a malignancy.

## 2. Case Presentation

An 83-year-old male with no prior gastrointestinal symptoms presented to our hospital with cough, nonbloody emesis, melena, dysphagia, and weight loss for the past month. He had a medical history of a myocardial infarction; he was an active smoker, drank alcohol occasionally, and denied illicit drug use. Patient had been hospitalized 6 weeks ago for pneumonia and acute kidney injury; at that admission, patient received IV antibiotics and a 1-week treatment of SPS for hyperkalemia. He did not continue to take the medication at home.

Physical examination revealed cachexia and tachycardia. The patient was ambulatory. Laboratory tests showed a normocytic anemia with hemoglobin of 9.3 g/dL, creatinine of 1.1, and hyperkalemia with potassium of 5.7 mEq/L.

**Figure 1:**
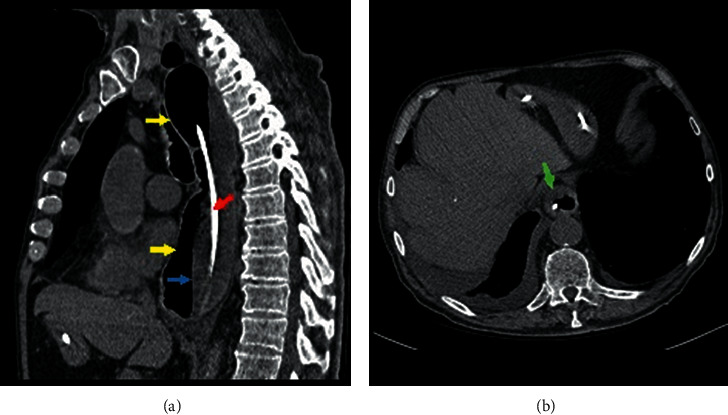
Computed tomography of the chest without IV contrast. Both sagittal (a) and axial (b) images demonstrating esophageal dilatation (yellow arrows) with an air-fluid level (blue arrow). There is a region of esophageal wall thickening near the gastroesophageal junction (green arrow) and an enteric tube (red arrow) is also noted.

Computed tomography of the chest without contrast (Figure 1) demonstrated a dilated esophagus with air-fluid level with an achalasia-like appearance and a mass in proximity to the gastroesophageal junction (GEJ) concerning for malignancy.

**Figure 2:**
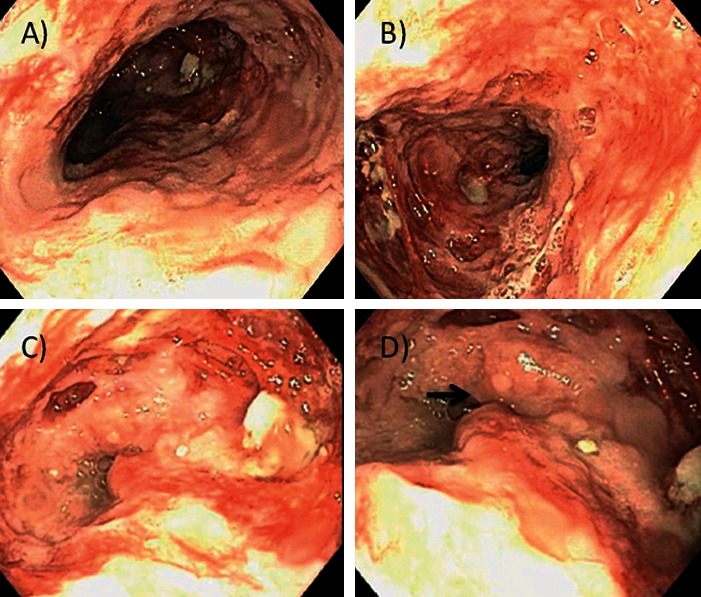
Esophagogastroduodenoscopy: esophageal mucosal lesions characterized by erythema and deep ulceration are noted in the middle (a and b) and distal (c and d) esophagus. A partially obstructing esophageal mass is seen in the distal esophagus (arrow).

Esophagogastroduodenoscopy (EGD) (Figure 2) showed an esophageal mucosa with erythema, congestion, deep ulcerations, and a mass in GEJ, which was partially obstructive. A nasogastric tube was introduced for nutritional support after multiple biopsies were taken of the friable mucosa and protruding mass.

**Figure 3:**
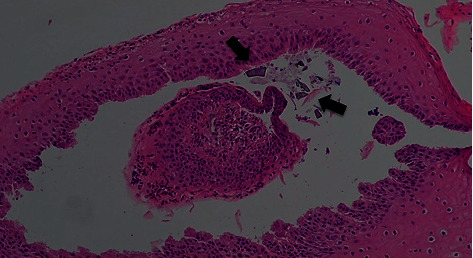
Esophageal biopsy: a mosaic pattern of basophilic rhomboid crystals (arrow) with surrounding areas of acute and chronic inflammation found mixed in the fragments of esophageal squamous mucosa. No malignancy is seen.

Pathology examination was consistent with fragments of squamous mucosa and basophilic rhomboid crystals with surrounding areas of mixed (acute and chronic) inflammation. The crystals arranged in a “mosaic pattern” were identified as SPS crystals. All biopsies examined were negative for any signs of metaplasia, dysplasia, or malignancy (Figure 3).

Proton pump inhibitor therapy and supportive management were initiated resulting in symptomatic improvement. After two weeks of nutritional support via the nasogastric tube, the patient was discharged tolerating oral intake. The patient refused follow-up EGD, but he remained asymptomatic at 4 months follow-up.

## 3. Discussion

We describe a case of SPS resin-induced severe esophagitis with endoscopic features similar to esophageal carcinoma. Only a few case reports have documented severe esophagitis in patients receiving SPS [1, 2, 7, 8]. Our patient was found to have an achalasia-like esophagus by imaging contributing to the retention of SPS in the esophagus, and an esophageal pseudotumor was found.

Medication-induced esophagitis may occur in the elderly population due to several factors including inadequate water intake with pills and recumbent positioning, polypharmacy, and decreased esophageal motility [6]. SPS is usually administered combined with sorbitol, a hypertonic agent that accelerates the transit through the small intestine and reduces the risk of obstruction and impaction. The colon is most commonly affected because sorbitol is metabolized by the normal microbiota, and mucosa is exposed longer to the resin, which may induce mucosal ischemia [5, 9–11]. Several direct and indirect mechanisms by which SPS may cause mucosal injury have been identified [4–6]. When the resin is in prolonged direct contact with the gastrointestinal lumen, an acute inflammatory response can occur in the epithelium [4]. The crystals directly elicit an area of inflammation usually after 24 hours of direct exposure. When SPS is administered with sorbitol as a combined formulation, sorbitol may cause a direct toxic effect by direct local prostaglandin activity in the site [4, 5]. Prolonged stasis of crystals in the lumen has the potential of developing erosions and ultimately necrosis [6]. The internalization of these crystals to the underlying intestinal mucosa with the combination of the inflammatory response may give an appearance of a luminal mass that can mimic a malignancy [4].

In the current literature, less than one-third of gastrointestinal complications due to SPS are localized in the upper GI tract, and the usual clinical presentation is abdominal pain, nausea, and melena [6, 12–14].

In a case series, the most common endoscopic findings of SPS-induced upper GI tract lesions were ulcerations and erosions [1]. Similar to our case, these lesions correlated with pathologic findings of SPS polygonal crystals adhered to the surface of the epithelium with surrounding inflammation [9]. SPS-induced colonic pseudotumors have been described. In all cases, the biopsies were negative for dysplasia with only SPS crystals and inflammation identified [4, 5, 15].

Treatment of SPS-induced mucosal injury is conservative with cessation of SPS. Resolution of inflammation usually occurs, once crystals are cleared from the GI tract. In some instances, the involvement of deep layers may cause irreversible damage due to failure of crystal clearance and prolonged ischemia of the submucosa and muscularis propria causing full thickness necrosis [16–18]. In these cases, surgical resection of the affected segment may be warranted [14, 19, 20].

In the presented case, the diagnosis of SPS-induced esophagitis was made based on SPS crystal identification with surrounding inflammation, without any other identifiable source of injury or malignancy. An underlying motility disorder could have contributed to SPS stasis in the esophagus, although no motility studies were performed. Additionally, lack of endoscopic follow-up prevents confirmation of esophagitis resolution. However, to support SPS-induced esophagitis diagnosis, several target biopsies were obtained and were negative for malignancy. SPS crystals with surrounding inflammation were consistently identified in affected lesions and not present in normal esophageal mucosa.

Once malignancy was ruled out, the achalasia-like syndrome in this patient placed a motility disorder high in our differentials as a possible cause of impaired clearance and accumulation of the resin in the esophagus. Limitations of our report are that an underlying motility disorder was not ruled out by manometry or barium esophagram and that follow-up endoscopy was not done to demonstrate resolution of the lesion.

SPS should be used with caution in the elderly population and in the presence of upper GI symptoms such as dysphagia, regurgitation, or abdominal pain and should be avoided in those with a known stricture or motility disorder. The endoscopic appearance of SPS-induced mucosal injury is nonspecific, and the diagnosis is based on the pathological hallmark of the mosaic pattern of rhomboid/triangular basophilic crystals with surrounding inflammation. In our case, extensive inflammation with ulcerations plus a mass-like thickening of the esophageal mucosa imitated a malignancy.

## Data Availability

The data used to support the findings of this study are included within the article.
